# Feasibility study of a sensor-to-segment calibration method to enhance upper limb motion analysis using an IMU-based system for clinical and home environments

**DOI:** 10.1371/journal.pone.0334177

**Published:** 2025-10-24

**Authors:** Alessandra Favata, Arnau Marzabal-Gatell, Josep M. Font-Llagunes, Rosa Pàmies-Vilà

**Affiliations:** 1 Department of Mechanical Engineering and Institute for Research and Innovation in Health (IRIS), Universitat Politècnica de Catalunya - BarcelonaTech (UPC), Barcelona, Spain; 2 Institut de Recerca Sant Joan de Déu, Esplugues de Llobregat, Spain; Polytechnic University of Marche: Universita Politecnica delle Marche, ITALY

## Abstract

Inertial Measurement Units (IMUs) represent a valid alternative to standard clinical assessment methods, such as clinical scales, for evaluating upper limb kinematics. A key aspect of utilizing IMUs effectively is ensuring precise sensor-to-segment calibration, which accounts for the relative orientation between the sensor and the attached body segment. This calibration is crucial to obtain accurate results. Although reliable calibration methods are available, their application in clinical and home environments remains challenging due to their complexity. This study aimed to validate a picture-based calibration method feasible for a clinical context and compare it against other standard methods. Ten healthy subjects performed daily activity tasks while upper limb kinematics was recorded using an optoelectronic motion capture system and an IMU-based system. Four calibration methods were compared using error metrics, including root mean square deviation (RMSD) and cross-correlation (XCORR). The results demonstrate that the proposed picture-based method provides highly accurate measurements for the first and second Euler rotation angles of the shoulder, with RMSD < 15^°^ and XCORR > 0.75 across most of the tasks. For the elbow joint, all calibration methods consistently yielded precise results for the first rotation (RMSD < 15^°^ and XCORR > 0.95) across the majority of tasks. The proposed sensor-to-segment calibration method improves the accuracy of upper limb motion data recorded with an IMU-based system compared to traditional methods. Moreover, the calibration approach is easy to use, making it suitable for clinical and home environments.

## Introduction

Functional movement assessment in patients with motor disorders is fundamental for evaluating disease progression and the effectiveness of treatments [1]. Traditional clinical evaluation methods, such as clinical scales, are often considered insufficiently accurate or objective for assessing motor function in patients with mobility impairment because they rely on the clinician’s observation. Additionally, clinical scales do not evaluate cognitive tasks within the patient’s habitual environment and do not take into account fluctuations in the patient’s symptoms over time. For these reasons, clinicians are becoming interested in analyzing objective movement data collected in both clinical and home scenarios [2–4]. Optoelectronic motion capture (OMC) systems are generally considered the gold standard in the motion analysis field but they are expensive, the post-processing is time consuming and not suitable for outside-laboratory analysis [5–7].

In this context, inertial measurement units (IMUs), electronic devices that track movement of the body where they are attached, offer a practical alternative for motor function assessments thanks to their small size, lightweight and the possibility to record data for long-term monitoring. Nevertheless, the signals measured by the IMU, attached to a body, are expressed in the sensor’s coordinate system. For this reason, a precise sensor-to-segment calibration is required to establish the relation between these two coordinate systems [8]. Misalignment and orientation inaccuracies during the calibration process can lead to errors in the data collected [9].

Several methods exist to establish the relative orientation between the body segment’s anatomical frame and the IMU reference frame. The most common calibration approaches are: assumed alignment (IMU-AA), functional alignment (IMU-FA), and augmented data (IMU-AD) [10]. However, each method has its limitations. IMU-AA depends on the clinician’s ability to place the sensors over the body segments, which can be challenging. Additionally, repeatability can be critical. IMU-FA requires the subjects to perform specific poses, making it unsuitable for patients with impaired mobility, that might not be able to perform these movements. Furthermore, these two calibration methods are not feasible particularly in case of patients equipped with medical apparatus that limit the sensor’s placement and joint range of motion. IMU-AD requires external tools, such as OMC systems or cameras, which reduces the simplicity and portability of IMU-based systems [10,11]. Additionally, in case the OMC is used as a calibrating device the accuracy of this method also depends on the markers’ placement. See Table S1 to compare the definition of these approaches with respect to the standard [12]. Bisi et al. obtained promising results using a device with a camera mounted on it to calibrate anatomical landmarks, of a synthetic femur, in the wearable sensor reference frame [13]. Another approach to improve the kinematic accuracy involves using an algorithm that accounts for physical joint or movement tasks constraints. This method is particularly applicable to hinge joints, such as the knee in the lower limb and the elbow in the upper limb, or when the movement task is predefined [14,15]. However, its effectiveness is limited to cases where a physical constraint can be applied to the joint or when the task is known in advance. This limitation poses challenges when applied to individuals with motor impairments, as their restricted or abnormal movement patterns may prevent the algorithm from correctly identifying and enforcing these constraints, thus reducing its reliability in such populations.

This study proposes a picture-based sensor-to-segment calibration method as an initial step toward achieving practical and accurate motion tracking using an IMU-based system for measuring upper limb kinematics for patients with motor impairments and for those equipped with medical devices (e.g., wheelchair), in both clinical and home settings. Compared to other alternatives, this method does not require the experience of a clinician to place the sensors or help patients to perform dynamic movements, facilitating its integration into real-world clinical applications where motion analysis is essential for tracking disease progression and evaluating interventions. In particular, the presented method relies on pictures taken while subjects maintain a static pose, requiring only a standard camera and a tripod. A preliminary evaluation of this calibration method has been carried out on a group of ten healthy volunteers to assess its feasibility and accuracy [12]. A secondary aim of this study includes the comparison of the proposed method with respect to three other standard calibration methods (IMU-AA, IMU-FA and IMU-AD) to evaluate the influence of the calibration methods on kinematic accuracy.

## Materials and methods

### Subjects

We analyzed ten healthy subjects (4 males, 6 females, age of 26.7 years ± 3.6, range 22-32, height 176 ± 1 cm, weight 73 ± 1 kg). The inclusion criteria were: (1) age between 20 years old and 35 years old, and (2) the ability to give their informed consent. The age range was limited to reduce variability in task performance due to age-related factors, thereby allowing us to focus on the primary effects under investigation.The only exclusion criterion was: (1) having any disease affecting the upper or lower limbs. The Ethics Committee of the UPC has issued a favorable opinion on the ethical aspects related to the research carried out in this project (Decision on November 17th, 2022, ID Code: 2022.7). The favorable approval of the application implies that the reviewed project complies with the criteria established by the institution’s own expertise. The recruitment of volunteers started on December 5th, 2022 and was completed on September 14th, 2023. All participants gave written informed consent.

### Equipment

Upper limb kinematics was recorded simultaneously with an OMC system and an IMU-based system, at the Biomechanical Engineering Lab (BIOMEC) of UPC.

The OMC system used twelve infrared cameras OptiTrack Flex V100:R2 (NaturalPoint Inc., USA) sampling at 100 Hz and utilized 15 passive reflective markers. These markers were placed on anatomical landmarks, according to previous works [16–18]. Marker trajectories were filtered with a 3rd-order low-pass Butterworth filter with a cut-off frequency of 5 Hz [19].

The IMUs were Xsens DOT sensors (Xsens Technologies, Netherlands). Xsens DOT is a wearable sensor (size: 36.3 × 30.4 × 10.8 mm (l × w × h); weight: 11.2 g) that incorporates a 3D accelerometer, gyroscope and magnetometer, with a sampling frequency of 60 Hz. The sensor fusion algorithm implemented to provide the 3D orientation, is an Xsens Kalman Filter core (XKFCore) [20]. The orientation output, in the form of a quaternion, was imported in Matlab (Matlab R2022b, The Mathworks, USA) to compute the kinematics.

The camera had a resolution of 1600 × 720 pixels and was mounted on a tripod. The pictures were taken positioning the tripod frontally and laterally, aligned with the anatomical planes.

### Movement task

**Fig 1:**
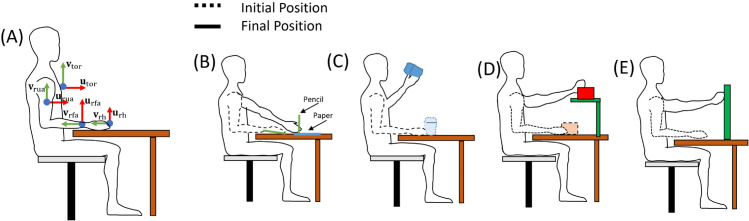
Body segment orientation (A). Graphical description of the four tasks performed: Draw (B); Drink (C); Move an object (D); and Unlock a locker (E).

The subjects were asked to perform three daily activity tasks (Drink, Move an object, and Unlock a locker) (see Fig 1). The ‘Drink’ task requires the subject to grasp a cup placed on the table, lift it, move it to the mouth and return it to the table. This activity primarily emphasizes the flexion and extension of the elbow. The ‘Move an object’ task entails grasping a box (30 × 20 × 20 cm), moving it in the horizontal plane, then placing it on a shelf and returning it to the table. This task underscores the importance of shoulder angles (rotation, elevation and axial rotation) along with the flexion and extension of the elbow. The task of ‘Unlock a locker’ involves grasping a key already inserted and rotating it clockwise and then anticlockwise. This task emphasizes the pronation and supination of the elbow and the movements of the wrist.

Additionally, we analyzed subjects performing a drawing task to study a movement used in clinical practice, such as clinical scales for patients with motor impairments. These four activities encompass a variety of actions and movements, showcasing the intricate coordination of multiple joints in the upper limb [21,22] (See Figure S1 and Table S2 in the supporting information). Ten repetitions of each task were performed. Ten trials were excluded due to the IMU-based system’s failure. The subjects were asked to perform the task at a comfortable speed. A 3-minute rest period was applied between repetitions.

### Biomechanical model

**Fig 2:**
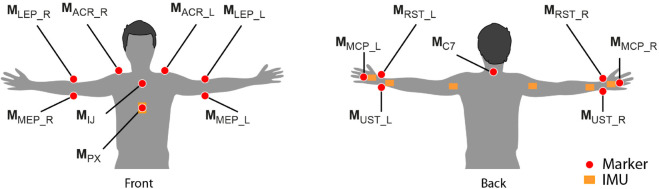
The retroreflective markers and inertial measurements units’ placement sites. Red circles represent the markers and the orange squares represent the sensors.

A segmental biomechanical model with seven rigid body segments was used to obtain the kinematics of the upper limb. All joints were considered as spherical joints, with rotational degrees of freedom (DoF) and no translational DoF. The model has 18 DoFs, 3 for each joint, since the movement of the scapula relative to the thorax and the humerus was neglected [11,16]. For both systems, seven body segments were identified: torso (*trs*), right upper arm (*rua*), left upper arm (*lua*), right forearm (*rfa*), left forearm (*lfa*), right hand (*rh*) and left hand (*lh*). Fig 2 shows markers and sensors placement for each body segment defined.

Markers on the trunk body segment were placed on the seventh spinal process (${𝐌}_{\text{C7}}$), deepest point of the incisura jugularis (${𝐌}_{\text{IJ}}$) and the processus xiphoideus (${𝐌}_{\text{PX}}$). Markers placed on the dorsal point on the acromioclavicular joint (${𝐌}_{\text{ACR}}$) and on the most caudal point on the lateral and medial epicondyle (${𝐌}_{\text{LEP}}$, ${𝐌}_{\text{MEP}}$) were used to define the upper arm segment, together with the middle point between ${𝐌}_{\text{LEP}}$ and ${𝐌}_{\text{MEP}}$, defined as ${𝐌}_{\text{M.Elb}}$. The latter, together with the middle point (${𝐌}_{\text{M.Wrs}}$) between the radial and ulnar styloid (${𝐌}_{\text{RST}}$, ${𝐌}_{\text{UST}}$), and the radial styloid (${𝐌}_{\text{RST}}$) were used to define the forearm segment. The hand segment was defined using ${𝐌}_{\text{M.Wrs}}$, ${𝐌}_{\text{RST}}$ and the 3rd metacarpal (${𝐌}_{\text{MCP}}$). Seven IMUs were placed on the upper limbs as follows: one sensor on the trunk, at the sternum level, one on the upper arm, centered on the lateral side of the proximal part, one on the forearm, at wrist level, and one on the dorsal surface of the hand. The model obtained from the position of the markers recorded with the OMC system was defined according to the ISB recommendations and previous works [16–18] (See File S1 of supporting information). The **u** represents the unit vector of the X-axis, directed anteriorly, the **v** represents the unit vector of the Y-axis, directed superiorly, and **w** represents the unit vector of the Z-axis, directed laterally to the right (see Fig 1).

The rotations were described using Euler angles: the Y-X’-Y” for the shoulder, the Z-X’-Y” for elbow and wrist, in accordance to the standards [16]. The X’-axis of the elbow and the Y”-axis of the wrist represent the floating axes of these joints, which are rarely reported. However, they have been included in this analysis to maintain consistency with the kinematic model defined, where all joints are represented as spherical joints.

### Sensor-to-segment calibration

The orientation of a joint between two adjacent body segments ($seg_{\text{i}}$, *i* = 1, ..., 7) can be expressed in quaternion form as follows [23,24]:

$${𝐪}_{seg_{\text{i}}}^{seg_{\text{i}-1}}={\left({𝐪}_{IMUseg_{\text{i}-1}}^{G}(t)⊗{𝐪}_{seg_{\text{i}-1}}^{IMUseg_{\text{i}-1}}\right)}^{-1}⊗{𝐪}_{IMUseg_{\text{i}}}^{G}(t)⊗{𝐪}_{seg_{\text{i}}}^{IMUseg_{\text{i}}}$$ (1)

where ${𝐪}_{IMUseg_{\text{i}-1}}^{G}$ and ${𝐪}_{IMUseg_{\text{i}}}^{G}$ represent the sensor orientation with respect to an earth-based global coordinate system (G) and are time-dependent (t); and ${𝐪}_{seg_{\text{i}-1}}^{IMUseg_{\text{i}-1}}$ and ${𝐪}_{seg_{\text{i}}}^{IMUseg_{\text{i}}}$ represent the relative orientation between the coordinate system of each body segment ($seg_{\text{i}}$, *i* = 1, ..., 7) and its corresponding IMU sensor ($IMUseg_{\text{i}}$). Thus, an initial calibration is needed to establish such relative orientation (${𝐪}_{seg_{\text{i}}}^{IMUseg_{\text{i}}}$, i = 1,...,7) which can be computed as follows:

$${𝐪}_{seg_{\text{i}}}^{IMUseg_{\text{i}}}={\left({𝐪}_{IMUseg_{\text{i}}}^{G}(t_{0})\right)}^{-1}⊗{𝐪}_{IMUref}^{G}(t_{0})⊗{𝐪}_{ref}^{IMUref}(t_{0})⊗{𝐪}_{seg_{\text{i}}}^{ref}(t_{0})$$ (2)

where ${𝐪}_{ref}^{IMUref}({\text{t}}_{0})$ represents the orientation of a body segment, used as reference (ref), with respect to its sensor (IMUref) at the initial calibration time ${\text{t}}_{0}$, and ${𝐪}_{seg_{\text{i}}}^{ref}$ represents the orientation of the analyzed segment ($seg_{\text{i}}$) with respect to the reference segment (ref) at calibration time. It is important to highlight that ${𝐪}_{seg_{\text{i}}}^{ref}$ will change according to the implemented calibration method. For instance, in the picture-based method this orientation will be obtained from the images. As it is possible to see in Eq (2), two quaternions (${𝐪}_{seg_{\text{i}}}^{IMUseg_{\text{i}}}$ and ${𝐪}_{ref}^{IMUref})$ are used to correct potential misalignment between sensors and their corresponding body segments and both of them are unknown. To determine one of these quaternions, the other must be known. For this reason, the sensor placed on the rua (IMUrua) was assumed to be perfectly aligned with the corresponding body segment (rua), meaning ${𝐪}_{rua}^{IMUrua}=1$. This assumption is commonly adopted in inertial-based upper-limb measurement systems [24]. In this work, the *rua* was chosen as reference segment (ref), as it was the segment where the sensor could be most easily aligned.

#### Standard calibration methods.

Three standard calibration methods were used for comparison with the one proposed in this work. Eq (1) applies to all calibrations, differing only on how ${𝐪}_{seg_{\text{i}}}^{ref}$ was obtained. As mentioned before, the traditional methods evaluated were:

- IMU-AA: The IMUs were placed on the body segments trying to align the IMU-fixed frame with the anatomical frame of the body segment, to which it is attached. In this approach, the assumption made is that these two reference frames (IMUs and body segment) were perfectly aligned. Therefore, no corrections were applied and ${𝐪}_{seg_{\text{i}}}^{ref}$ was considered equivalent to the identity, for all the body segments.
- IMU-FA: Subjects were asked to perform a static pose with the arm fully extended, the elbow flexed at 90^°^, and the wrist aligned with the elbow. In this case, the assumption made was that the pose was perfectly performed. Therefore, ${𝐪}_{seg_{\text{i}}}^{ref}$ was set to represent the orientation of the static pose for each body segment.
- IMU-AD: To apply this method, the information provided from the OMC was used to determine the relative orientation between the sensor and the body segment. Thus, ${𝐪}_{seg_{\text{i}}}^{ref}$ was obtained from the OMC data at time calibration $\left(t_{0}\right)$.

#### Proposed picture-based calibration method.

In this study, a straightforward picture-based sensor-to-segment calibration (IMU-PIC) was implemented; it can be easily applied in clinical or home settings. The quaternion ${𝐪}_{seg_{\text{i}}}^{ref}$ was the estimated orientation obtained from the images, as explained in the following procedure.

**Fig 3:**
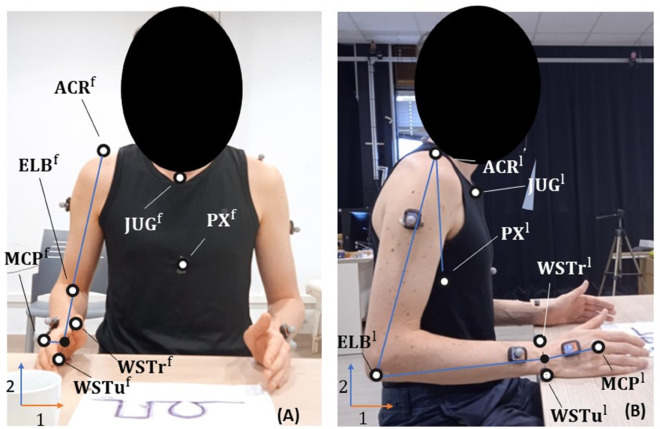
Static pose performed during the calibration process from (A) frontal view and (B) lateral view, where PX corresponds to processus xiphoideus, JUG the incisura jugularis, ACR the acromion, ELB the middle point between the two epicondyles, WSTr the radial styloid, WSTu the ulnar styloid and MCP the 3rd metacarpal.

Before starting the task, the subjects were asked to remain still in a comfortable pose for 5 seconds, as shown in Fig 3, during which images from both the frontal and lateral planes were taken.

Our method requires obtaining the joint angles at $t_{0}$ using the calibration images and integrating this information with the IMUs data. Fig 3 shows the points selected to define the body segments.

Each point in the pictures was defined by two coordinates. The points in the lateral picture were represented as ${𝐏}^{l}=[P_{1}^{l},P_{2}^{l}{]}^{⊤}$, where $P_{1}^{l}$ and $P_{2}^{l}$ represent the horizontal and vertical coordinates of the picture, respectively. Similarly, the points in the frontal picture were defined as ${𝐏}^{f}=[P_{1}^{f},P_{2}^{f}{]}^{⊤}$, where $P_{1}^{f}$ and $P_{2}^{f}$ denote the horizontal and vertical coordinates of the frontal view (see Fig 3). To address the issue of varying distances from which the images were captured, the coordinates obtained from the frontal images were scaled by a factor of *s*. This factor was calculated as follows:

$$s=\frac{{JUG}_{2}^{l}-{PX}_{2}^{l}}{{JUG}_{2}^{f}-{PX}_{2}^{f}}$$ (3)

Then, for a general point **P**, the 3D Cartesian coordinates expression as a function of the picture coordinates was taken as:

$$𝐏=[P_{1}^{l},P_{2}^{l},-sP_{1}^{f}{]}^{⊤}$$ (4)

where the minus sign was added to overcome the issue of the 1-axis of the frontal picture that has opposite direction with respect to the Z-axis of the global reference frame. To obtain the orientation of the segments let ${\tilde{𝐮}}_{seg}$, ${\tilde{𝐯}}_{seg}$, ${\tilde{𝐰}}_{seg}$ be the unit vectors of the local coordinate system for segment *seg*. The expression of $\tilde{𝐮}$, $\tilde{𝐯}$, $\tilde{𝐰}$, as a function of the points defined, was shown in Table 1, where processus xiphoideus (PX), the incisura jugularis ( **JUG**), the acromion ( **ACR**), the middle point ( **ELB**) between the ulnar ( **WSTu**) and radial styloid ( **WSTr**), and the 3rd metacarpal ( **MCP**) were *R*^3^ points defined as in Eq (4) and $\tilde{𝐳}$ was the Z-axis of the global frame. Even if the ACR position does not correspond to the point suggested by the standard, the landmark as described above was chosen because it was easily recognizable. All the points were manually selected by the same operator.

**Table 1:** Definition of the body segment coordinate systems for the right side with the data obtained from the the pictures, where PX corresponds to processus xiphoideus, JUG the incisura jugularis, ACR the acromion, ELB the middle point between the two epicondyle, WSTr the radial styloid, WST the middle point between WSTr and WSTu and MCP the 3rd metacarpal.

Segment	Origin	$\tilde{𝐮}$	$\tilde{𝐯}$	$\tilde{𝐰}$
trs	**PX**	$\frac{ACR-PX}{\left||ACR-PX|\right|}\times {\tilde{𝐰}}_{tor}$	${\tilde{𝐰}}_{tor}\times {\tilde{𝐮}}_{tor}$	$\tilde{𝐳}$
rua	**ELB**	${\tilde{𝐯}}_{rua}\times {\tilde{𝐰}}_{rua}$	$\frac{ACR-ELB}{\left||ACR-ELB|\right|}$	${\tilde{𝐯}}_{rua}\times {\tilde{𝐯}}_{rfa}$
rfa	**WST**	${\tilde{𝐯}}_{rfa}\times {\tilde{𝐰}}_{rfa}$	$\frac{ELB-WST}{\left||ELB-WST|\right|}$	$\frac{WSTr-WST}{\left||WSTr-WST|\right|}\times {\tilde{𝐯}}_{rfa}$
rh	**MCP**	${\tilde{𝐯}}_{rh}\times {\tilde{𝐰}}_{rh}$	$\frac{WST-MCP}{\left||WST-MCP|\right|}$	$\frac{WSTr-MCP}{\left||WSTr-MCP|\right|}\times {\tilde{𝐯}}_{rh}$

To define ${\tilde{𝐰}}_{rua}$, the lateral epicondyle, which was not easily identifiable through the picture, has to be selected. To address this issue, ${\tilde{𝐰}}_{rua}$ was obtained as the normal vector to the plane defined by ${\tilde{𝐯}}_{rua}$ and ${\tilde{𝐯}}_{rfa}$.

Since vectors $\tilde{𝐮}$, $\tilde{𝐯}$, $\tilde{𝐰}$ were expressed in the global frame (G), the rotation matrix ${𝐑}_{G}^{seg}=[{\tilde{𝐮}}_{seg}\;{\tilde{𝐯}}_{seg}\;{\tilde{𝐰}}_{seg}]$ defines the orientation of the segment with respect to the global reference frame. To obtain the relative orientation between the segments, the following transformation was required to obtain the desired rotation matrices:

$${𝐑}_{rua}^{tor}=({𝐑}_{tor}^{G}{)}^{⊤}{𝐑}_{rua}^{G},\;\;\;{𝐑}_{rfa}^{rua}=({𝐑}_{rua}^{G}{)}^{⊤}{𝐑}_{rfa}^{G},\;\;\;{𝐑}_{rh}^{rfa}=({𝐑}_{rfa}^{G}{)}^{⊤}{𝐑}_{rh}^{G}$$ (5)

Finally, the Euler angles were obtained at time of calibration from the rotation matrices (see Eqs (1) and (2) in File S1 of supporting information).

Although this method may seem similar to IMU-AA, there are key differences. IMU-AA requires perfect alignment of all sensors with body segments, whereas IMU-PIC only requires one sensor to be perfectly aligned, simplifying the setup and enhancing accuracy, which will be demonstrated and commented in the following sections (Results and Discussion).

### Statistical analysis

Angular errors (Δ=α(IMU) − α(OMC)) across all axes were calculated at the calibration time. To evaluate kinematics accuracy, Root Mean Square Deviation (RMSD), as a measurement of disagreement, and cross-correlation (XCORR), as measurement of agreement were employed [11,12,25–27]. Levels of agreement were considered poor when XCORR < 0.45, fair when 0.45 < XCORR < 0.75 and high when XCORR > 0.75. In addition, being an *in vivo* study, RMSD up to 15^°^ was considered acceptable [2]. To assess the sole impact of the initial offset on the kinematics, the RMSD and XCORR of the calibration methods IMU-AA, IMU-FA and IMU-PIC were compared against IMU-AD. IMU-AD serves as reference because it represents the best estimation of the initial pose. Normal distribution was tested using the Kolmogorov-Smirnov test. Group differences were assessed based on data distribution, utilizing Kruskal-Wallis or ANOVA, followed by Wilcoxon signed rank sum or paired-sample t-test. The level of significance was set at 0.05. Statistical analyses were performed with built-in Matlab functions (Matlab R2022b, The Mathworks, USA).

## Results

### Offset at calibration time

**Fig 4:**
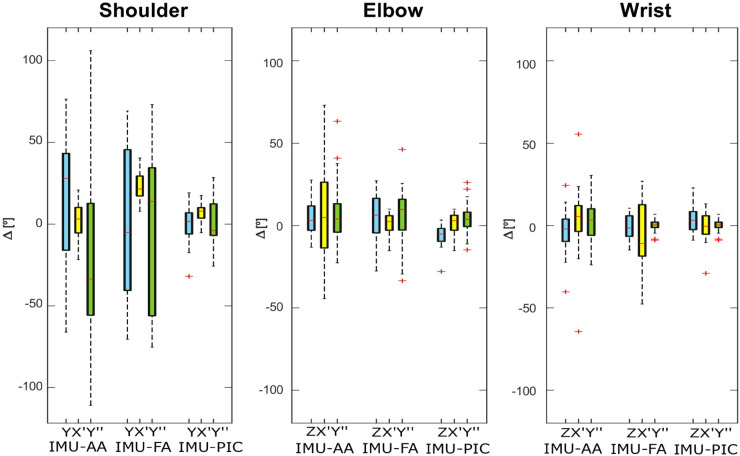
Boxplots represent the variation in angle values (*Δ*) between the analyzed calibration methods (IMU-AA, IMU-FA and IMU-PIC) and the corresponding OMC value, for each subject across different tasks. Red ‘+’ symbols indicate outliers. The color legend represents the axis of rotation for each joint: blue for the first axis, yellow for the second, and green for the third one.

Fig 4 presents boxplots depicting the offset (Δ) in joint angles at calibration time between each analyzed method (IMU-AA, IMU-FA and IMU-PIC) and the OMC values, with errors grouped by calibration method, with consistent colors across the axes of rotation. Lower *Δ* values indicate good accuracy, as the discrepancy reflects the differences against the OMC.

Higher interquartile ranges were evident particularly for IMU-AA and IMU-FA compared to IMU-PIC, particularly in the shoulder joint. For the elbow joint, similar errors were found across all methods but the standard calibration methods (IMU-AA and IMU-FA) showed higher interquartile range. For the wrist joint, IMU-PIC showed the lowest error and interquartile range compared to IMU-AA and IMU-FA.

### Upper limb joint kinematics

Table 2 shows the median (interquartile range) values of XCORR and RMSD for each calibration method (IMU-AA, IMU-FA, IMU-AD and IMU-PIC) with respect to the OMC. To evaluate the sole effect of the calibration on the kinematics, in Table 2 we highlighted in orange the values that were statistically significantly different from IMU-AD. Additionally, to facilitate the comparison with the gold standard, the RMSD was also computed, for each task analyzed, after removing the offset at time calibration. This analysis allows a clearer evaluation of waveform similarity and aligns with previous validation approaches. As expected, RMSD values decreased. The findings indicate that IMU-AD and IMU-PIC exhibit the lowest discrepancies, while IMU-AA and -IMU-FA present higher errors. These findings confirm the trends reported in Table 2 and are detailed in the Supporting Information (See File S2 of supporting information).

**Table 2:** Measures of agreement (XCORR) and disagreement (RMSD) between the OMC and each calibration method (IMU-AA, IMU-FA, IMU-AD and IMU-PIC) analyzed, median (interquartile range) of the dominant hand. Y-axis, X’-axis, Y”-axis, Z-axis, X’-axis, Y”-axis represent the angle around of this axis. Statistically significant differences with respect to the IMU-AD were highlighted in orange.

		Shoulder			Elbow			Wrist		
	XCORR	Y-axis	X’-axis	Y”-axis	Z-axis	X’-axis	Y”-axis	Z-axis	X’-axis	Y”-axis
	IMU-AA	0.958 (0.52)	0.977 (0.04)	0.974 (0.05)	0.998 (0)	–0.512 (1.55)	0.438 (0.6)	0.712 (0.3)	0.971 (0.04)	0.298 (1.22)
	IMU-FA	0.835 (0.19)	0.961 (0.07)	0.874 (0.28)	0.998 (0.01)	0.53 (0.8)	0.464 (0.83)	0.372 (1.33)	0.716 (1.02)	0.171 (0.85)
	IMU-AD	0.988 (0.01)	0.991 (0.01)	0.985 (0.01)	0.998 (0.01)	0.549 (0.73)	0.916 (0.7)	0.81 (0.27)	0.977 (0.04)	0.486 (0.92)
	IMU-PIC	0.989 (0.02)	0.989 (0.03)	0.976 (0.05)	0.998 (0.01)	0.374 (1.24)	0.78 (0.75)	0.435 (1.05)	0.968 (0.03)	–0.18 (0.83)
	RMSD (deg)	Y-axis	X’-axis	Y”-axis	Z-axis	X’-axis	Y”-axis	Z-axis	X’-axis	Y”-axis
	IMU-AA	32.02 (37.97)	9.15 (6.4)	32.7 (30.4)	11.12 (9.09)	23.12 (19.44)	14.85 (7.99)	8.7 (6.68)	12.83 (15.32)	8.05 (12.07)
	IMU-FA	42.63 (19.66)	16.56 (10.24)	46.71 (12.89)	11.45 (5.9)	10.03 (9.14)	19.04 (17.31)	10 (11.23)	15.79 (19.81)	6.86 (3.7)
	IMU-AD	12.23 (7.79)	7.13 (6.22)	16.35 (7.91)	6.19 (8.8)	8.43 (4.27)	14.18 (12.28)	4.24 (11.2)	4.5 (5.28)	4.37 (3.76)
-10*Draw	IMU-PIC	9.36 (10.77)	9.24 (5.79)	18.73 (5.04)	11.9 (11.47)	9.44 (3.48)	20.1 (14.43)	9.71 (10.09)	9.21 (4.66)	6.55 (5.93)
	XCORR	Y-axis	X’-axis	Y”-axis	Z-axis	X’-axis	Y”-axis	Z-axis	X’-axis	Y”-axis
	IMU-AA	0.941 (0.2)	0.99 (0.02)	0.959 (0.02)	0.996 (0.01)	–0.133 (1.22)	0.507 (1.12)	0.709 (0.23)	0.935 (0.06)	–0.154 (0.74)
	IMU-FA	0.84 (0.34)	0.951 (0.04)	0.764 (0.34)	0.995 (0.01)	–0.347 (0.6)	–0.191 (0.9)	0.694 (0.21)	0.171 (0.75)	0.031 (0.41)
	IMU-AD	0.982 (0.01)	0.994 (0.01)	0.982 (0.03)	0.995 (0.01)	0.257 (0.89)	0.721 (0.8)	0.717 (0.25)	0.932 (0.09)	0.126 (0.5)
	IMU-PIC	0.983 (0.01)	0.991 (0.02)	0.972 (0.03)	0.995 (0.02)	–0.099 (0.68)	0.23 (0.9)	0.717 (0.22)	0.926 (0.08)	0.025 (0.49)
	RMSD (deg)	Y-axis	X’-axis	Y”-axis	Z-axis	X’-axis	Y”-axis	Z-axis	X’-axis	Y”-axis
	IMU-AA	38.84 (23.22)	9.67 (8.49)	40.19 (27.17)	13.56 (5.64)	24.64 (22.66)	25.91 (18.7)	10.51 (19.56)	14.08 (21.06)	14.25 (26.16)
	IMU-FA	51.45 (42.84)	16.74 (10.28)	64.6 (41.55)	16.3 (11.58)	14.51 (7.01)	31.37 (13.67)	13.21 (12.79)	15.31 (7.51)	13.1 (4.44)
	IMU-AD	14.28 (3.56)	6.32 (5.3)	12.2 (18.46)	12.3 (10.09)	8.93 (9.05)	17.36 (11.36)	12.68 (9.65)	7.01 (4.32)	11.73 (5.84)
Drink	IMU-PIC	19.43 (15.1)	6.74 (10.97)	22.14 (23.54)	16.24 (9.76)	11.66 (9.82)	24.45 (12.74)	12.29 (9.08)	11.25 (7.13)	12.24 (4.87)
	XCORR	Y-axis	X’-axis	Y”-axis	Z-axis	X’-axis	Y”-axis	Z-axis	X’-axis	Y”-axis
	IMU-AA	0.98 (0.1)	0.99 (0.01)	0.92 (0.15)	0.993 (0.02)	0.324 (0.92)	0.925 (0.13)	0.641 (0.6)	0.964 (0.02)	0.711 (0.72)
	IMU-FA	0.92 (0.04)	0.971 (0.04)	0.843 (0.14)	0.99 (0.02)	0.297 (0.8)	0.937 (0.13)	0.468 (1.17)	0.857 (0.62)	0.65 (1.04)
	IMU-AD	0.995 (0.01)	0.995 (0.01)	0.983 (0.02)	0.99 (0.02)	0.517 (0.3)	0.938 (0.12)	0.725 (0.81)	0.965 (0.07)	0.687 (1.11)
	IMU-PIC	0.994 (0.01)	0.994 (0.01)	0.981 (0.04)	0.992 (0.02)	0.365 (0.79)	0.957 (0.08)	0.447 (1.21)	0.956 (0.09)	0.652 (1.07)
	RMSD (deg)	Y-axis	X’-axis	Y”-axis	Z-axis	X’-axis	Y”-axis	Z-axis	X’-axis	Y”-axis
	IMU-AA	32.23 (14.52)	11.34 (4.76)	33.08 (52.07)	12.85 (14.12)	17.04 (11.07)	21.03 (10.46)	15.05 (8.39)	10.67 (5.92)	17.06 (9.69)
	IMU-FA	37.68 (9.14)	17.9 (17.88)	42.31 (19.66)	18.89 (10.47)	14.5 (3.62)	27 (7.6)	20.18 (15.33)	22.95 (9.42)	20.19 (15.51)
	IMU-AD	12.82 (8.19)	10.92 (6.14)	20.25 (7.19)	20.5 (18.41)	13.39 (4.27)	22.42 (20.91)	10.39 (15.52)	10.71 (4.4)	22.13 (18.36)
Move an object	IMU-PIC	13.19 (11.9)	10.29 (7.47)	20.84 (20.68)	21.7 (13.05)	13.88 (5.83)	22.39 (12.96)	13.35 (13.59)	14.53 (11.56)	28.2 (18.19)
	XCORR	Y-axis	X’-axis	Y”-axis	Z-axis	X’-axis	Y”-axis	Z-axis	X’-axis	Y”-axis
	IMU-AA	0.92 (0.28)	0.992 (0.02)	0.968 (0.41)	0.985 (0.06)	0.123 (0.67)	0.812 (0.18)	0.792 (0.48)	0.969 (0.05)	0.348 (0.72)
	IMU-FA	0.912 (0.12)	0.973 (0.02)	0.866 (0.32)	0.987 (0.02)	0.153 (0.24)	0.674 (0.27)	0.878 (0.4)	0.64 (0.68)	0.4 (0.51)
	IMU-AD	0.983 (0.02)	0.996 (0)	0.975 (0.04)	0.99 (0.02)	0.586 (0.32)	0.809 (0.24)	0.929 (0.24)	0.97 (0.1)	0.467 (0.45)
	IMU-PIC	0.983 (0.02)	0.994 (0)	0.97 (0.07)	0.989 (0.01)	0.229 (0.77)	0.749 (0.39)	0.887 (0.31)	0.963 (0.07)	0.338 (0.42)
	RMSD (deg)	Y-axis	X’-axis	Y”-axis	Z-axis	X’-axis	Y”-axis	Z-axis	X’-axis	Y”-axis
	IMU-AA	26.21 (50.13)	10.51 (14.77)	42.05 (40.87)	15.61 (15.52)	27.1 (20.37)	23.37 (12.2)	15.77 (8.97)	10.29 (4.24)	16.73 (4.9)
	IMU-FA	38.24 (15.38)	21.7 (10.28)	44.29 (15.38)	16.37 (14.97)	14.37 (11.52)	25.91 (15.22)	11.4 (10.79)	17.88 (12.44)	15.37 (7.55)
	IMU-AD	13.78 (10.39)	8.34 (4.57)	18.68 (9.11)	9.37 (8.89)	11.91 (11.47)	23.04 (15.72)	9.19 (9.26)	5.54 (6.25)	15.11 (5.57)
Unlock a locker	IMU-PIC	14.04 (10.98)	11.04 (8.9)	25.68 (20.58)	13.91 (8.82)	13.68 (10.14)	29.21 (20.28)	13.77 (8.82)	7.4 (5.44)	17.39 (5.5)

**Fig 5:**
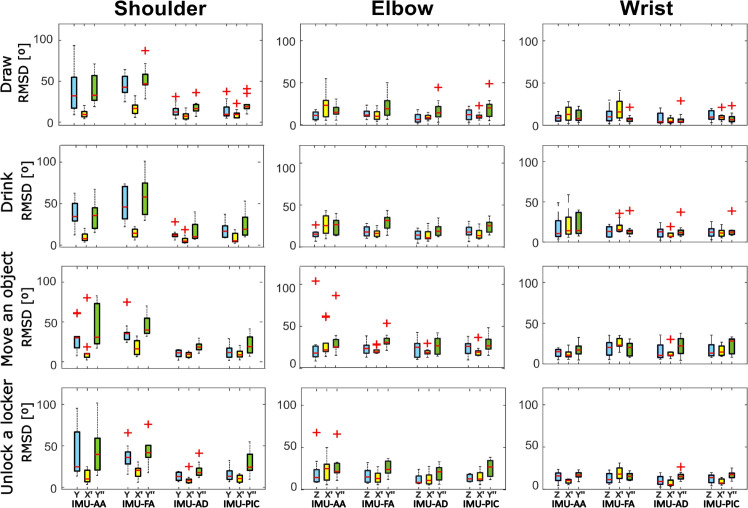
The variation RMSD between the analyzed calibration methods (IMU-AA, IMU-FA, IMU-AD and IMU-PIC) and the corresponding OMC value for each subject across different tasks. Red ‘+’ symbols indicate outliers. The color legend represents the axis of rotation for each joint: blue for the first axis, yellow for the second, and green for the third one.

In Fig 5 it is possible to notice high interquartile ranges for IMU-AA and IMU-FA across all the tasks, nearly twice the ones of the IMU-AD. Instead, IMU-PIC led to results close to those of IMU-AD.

**Fig 6:**
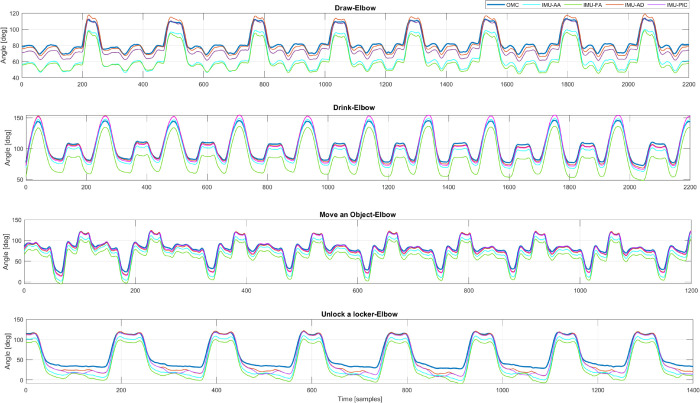
Comparison of Z-axis elbow angles trajectories between the reference (OMC) and the IMU-based system calibrated with the four calibration methods (IMU-AA, IMU-FA, IMU-AD and IMU-PIC) when drawing, drinking, moving an object and unlocking a locker.

The elbow angle trajectories of a participant’s trial were shown in Fig 6. It is possible to observe that IMU-AD and IMU-PIC align closely with the reference (OMC), while IMU-AA and IMU-FA do not. This joint has been chosen just as a representative example and the same considerations may be applied to the others.

For shoulder angles, IMU-AD and IMU-PIC both led to accurate kinematic results, with high cross-correlation (XCORR > 0.95) and low discrepancy (RMSD < 15^°^), exception made for the Y”-axis.

Interestingly, the angle of Z-axis of the elbow showed across all the calibration methods an high cross-correlation. The best results, in terms of RMSD, were obtained for the ‘Draw’ and for the ‘Unlock a locker’ tasks. The angle of Y”-axis of the elbow, instead, shows high discrepancy across all the calibration methods analyzed.

For the wrist joint, the Z-axis angle shows low discrepancy (RMSD < 15^°^) and high correlation (XCORR > 0.75) for the ‘Unlock a locker’ task with both IMU-AD and IMU-PIC. The X’-axis angle of the wrist also displays low discrepancy (RMSD < 15^°^) and high correlation (XCORR > 0.75) across all tasks for both IMU-AD and IMU-PIC. Additionally, although a statistically significant difference between IMU-AD and IMU-PIC was observed in the ‘Drawing’ task, IMU-PIC and OMC still exhibited low disagreement (RMSD < 10^°^) and high correlation (XCORR > 0.95).

## Discussion

The aim of this study was to introduce a practical yet accurate calibration method to improve IMU-based upper limb motion analysis for use in both clinical and home environments. The findings of this study indicate that the proposed IMU-PIC method is accurate, as no statistical difference was found between IMU-PIC and IMU-AD, which was used as the benchmark. Furthermore, it is easy to implement, as it required few tools, and it is feasible for patients with motor impairments, as it does not require the performance of complex poses. A previous study by Bisi et al. proposed a camera-based calibration device for anatomical landmark identification [13]. Although their approach led to promising results, some limitations must be considered. Firstly, the method requires a complex calibrating device. Additionally, the pattern used for camera calibration, which is attached to the sensor, must be calibrated with an OMC system. Moreover, to ensure method robustness, several images are needed. Finally, this method was tested only on a synthetic femur, overlooking the difficulties of *in vivo* experiment. By contrast, our method offers a practical approach, despite some limitations, to record kinematic data outside an equipped laboratory with patients with motor impairments.

Regarding the offset at calibration time, according to our results IMU-PIC appears to be the best option compared to IMU-AA and IMU-FA. Regarding IMU-AA, despite efforts to align the coordinate system of the sensors with the one of the body segments, inherent challenges caused deviations from the ground truth. Caruso et al. addressed this issue by incorporating physiological joint limits and task-specific motor characteristics into their optimization process, which helped mitigate sensor orientation drift over prolonged recordings. Their method was specifically applied to a controlled task (i.e., drawing on paper) where the movement was well-defined, allowing them to optimize joint angle estimations using constraints based on the expected motor patterns [14]. Nevertheless, this approach is not applicable in the case of a calibration method that enhances kinematic accuracy across various movements, rather than focusing on a single predefined task, making task-specific constraints impractical. Similarly, Digo et al. implemented an anatomical calibration for real-time estimation of upper limb kinematics during a task of moving a box. To reduce the error between the sensors and the gold standard, they removed the offset between these two systems. The errors obtained were very low [28]. Unfortunately, this adjustment cannot be made in a clinical or home setting where there is no data available to use as reference. Similarly in case of IMU-FA, despite participants’ attempts to achieving the desired static poses, difficulties persisted leading to discrepancies. For instance, IMU-FA calibration presented high disagreement for the X’-axis, due to the impossibility of having a shoulder elevation equal to zero at calibration time [29]. Robert-Lanchaine et al. reported initial offset errors between 5^°^ and 15^°^ for static pose calibration, which aligns with our findings for the elbow and wrist [29]. We observed higher values for the shoulder, likely due to pose differences. Bonfiglio et al. examined the offset between IMU-based system and OMC for the elbow joint, finding similar values to our study [30]. We believe that understanding the relation between initial offset and recorded kinematics is fundamental for obtaining reliable data for clinical analysis.

IMU-PIC showed shoulder kinematic accuracy comparable to IMU-AD, with no statistically significant difference observed. This finding is particularly important, as the shoulder is the initial joint in the kinematic chain. Instead, IMU-AA and IMU-FA led to high disagreement with respect to OMC, as expected due to the high offset at calibration time. Regarding the angle around the Y-axis and Y”-axis of the shoulder, Hoglund et al. studied the effect of the sensor’s placement in assessing the upper limb kinematics. They highlighted the positioning of the sensor on the upper arm due to soft tissue interference, suggesting that a distal placement would be preferable to a more proximal placement [31]. For the Y”-axis of the elbow, high RMSD was found across all the methods. Nevertheless, in a subgroup of subjects, IMU-PIC showed low disagreement (RMSD < 10^°^) suggesting that discrepancies may be related to the OMC signal or sensor positioning issues along the forearm. In [31], the authors also emphasized the importance of sensor placement on the forearm due to anatomical constraints, resulting in larger rotation at the distal end of the forearm. The best results for the wrist angle around the Z-axis were achieved during the "Unlock a Locker" task, likely because this axis was more actively involved in the execution of this specific task. Accurate results were also achieved for the angle around the X’-axis across all the tasks proving the validity of the IMU-based system for this specific angle. It is worth underlining that low and negative correlations were found for the X’-axis of the elbow and Y”-axis of the wrist. These two axes exhibited low ROM (as shown in Table S3 of supplementary materials), supporting the common assumption that the elbow and wrist might be modeled as hinge joints. Due to the limited movement in these axes, the signal quality was low, making comparisons difficult and ineffective.

The kinematic analysis revealed relatively high RMSD compared to previous research, likely due to the complexity of the movements involved, which can increase errors [24,25]. It is worth underlining that, as expected, removing the offset between the OMC and the IMU data, caused a decrease of the RMSD for almost all the methods (Supplementary Information File S2). Additionally, the joint range of movement involved in each specific task may affect kinematic data accuracy [24,31–33]. Therefore, the ROM for each joint and task was reported, using OMC signal (see Table S3 in Supporting information). A previous work analyzed the upper limb kinematics during a ‘Drawing’ task [24]. RMSD values, comparable to the ones obtained with IMU-AD and IMU-PIC, were reported for shoulder kinematics. In [24], lower errors were found for elbow flexion, which may be due to differences in drawing path or elbow ROM. It is worth underlining that errors up to 15^°^ for the elbow were considered an acceptable level of accuracy in case of *in vivo* studies [2]. Bonfiglio et al. analyzed the RMSD of drinking tasks and obtained results comparable to ours, and noted that errors increase when transitioning from pure elbow flexion to multi-joint tasks [30]. In [25], the authors analyzed a task of handling a box and they found lower errors with respect to the ones shown here. These discrepancies may be attributed to variations in the task, leading to different movement complexity. Similar results were found in [30] when analyzing a ‘Box off Shelf’ task for the elbow joint. To our knowledge, no previous studies compared an IMU-based system and the OMC for the ‘Unlock a locker’ task.

Limitations of this study include potential soft tissue artifacts and anatomical landmark identification affecting the OMC system used as a reference. Despite this, OMC remains a valid reference [25,29]. To make the picture-based method easy to perform, we assumed a constant camera depth and did not account for the camera’s intrinsic or extrinsic parameters as Bisi et al. did, which may introduce errors [13]. We defined the lateral and frontal planes on the subject’s anatomical planes and tried to align them as accurately as possible without specific equipment. The choice of the sensor placed on the upper arm was driven partly by practical considerations (i.e., participant comfort and experimental feasibility); future studies should explore alternative placements, such as the forearm or torso. Despite these approximations, the accuracy of IMU-PIC was consistent with previous studies and even surpassed common calibration methods. Another limitation of this work is the manual selection of the anatomical landmarks from the images. Future work should implement 3D pose-estimator software, based on deep learning such as MediaPipe or Azure Kinect, to automatically obtain the landmarks’ 3D information from the images, to enhance practicality and accuracy (e.g., selecting the correct shoulder joint position). Moreover, the estimation of the shoulder joint center could be enhanced by the use of regression equations [34].

## Conclusion

This study underscores the importance of the sensor-to-segment calibration to improve IMU-based upper limb motion capture and the potential clinical utility of the proposed calibration method, especially for patients with motor impairments. The picture-based method offers a promising yet accurate alternative that could enhance the accessibility and usability of motion capture technology in clinical and home settings, ultimately benefiting patients with motor impairments. Finally, future research should test the validity of this method in a group of participants with motor disorders.

## Supporting information

S1 Fig Drawing path: Trajectory that subjects had to follow used in the task ‘Drawing’. (TIFF)

S1 Table ISB calibration terms: Terminology for the calibration approaches in the ISB guidelines and in the present work. (PDF)

S2 Table Task description: Detailed description of the tasks performed and the joint angles analyzed. (PDF)

S1 File Biomechanical model: Detailed description of the definition of the body segment with the optoelectronic motion capture. Additionally, the rotation matrices used to define the angular position of the joint from the images were defined.
(PDF)

S2 File Additional data analysis: RMSD analysis for the four tasks, highlighting the influence of offset between IMU and OMC data on kinematic accuracy. (PDF)

S3 Table Biomechanical results: Analysis of shoulder, elbow, and wrist range of motion across the four evaluated tasks. (PDF)
