# Supplementary figures and images for: Feasibility study of a sensor-to-segment calibration method to enhance upper limb motion analysis using an IMU-based system for clinical and home environments
Source: PLoS One. 2025 Oct 24;20(10):e0334177. doi: 10.1371/journal.pone.0334177 (PMC12551884; doi:10.1371/journal.pone.0334177)

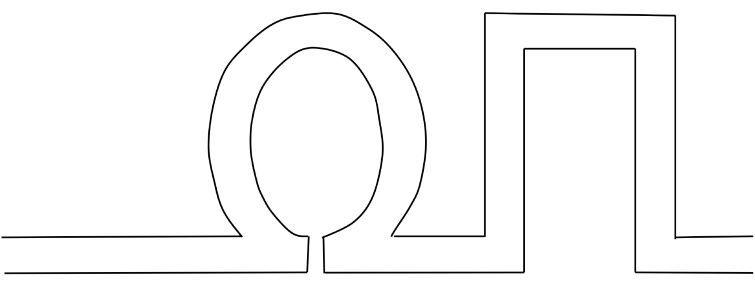

Supplement: S1 Fig — (TIFF) [file pone.0334177.s001.tiff]
