# Supplementary material for: Feasibility study of a sensor-to-segment calibration method to enhance upper limb motion analysis using an IMU-based system for clinical and home environments
Source: PLoS One. 2025 Oct 24;20(10):e0334177. doi: 10.1371/journal.pone.0334177 (PMC12551884; doi:10.1371/journal.pone.0334177)
Supplement: S1 Table — (PDF) [file pone.0334177.s002.pdf]

Table 1. Terminology used to define the calibration approach used in the ISB guidelines and in the present work

ISB GUIDELINES	PRESENT WORK
Manual Unit Alignment: The simplest solution for axes identification is to manually align the geometrical axes of the IMU housing with the axes of the anatomical coordinate system	Assumed alignment: The IMU was attached to the body segment trying to align IMU frame with the anatomical frame
Anatomical landmark identification approach: using a calibration device	Augmented data: using the stereophotogrammetric system as calibration device
	Picture-based alignment: using a camera as calibration device
Functional approach: use joint motion to identify the relevant axes of rotation	Functional Alignment*: participants perform movement(s) or pose(s) for which at least one anatomical axes can be estimated<br><br>*In our approach, due to the mobility impairment of the final user, this method requires to perform just static pose
