# Supplementary material for: Feasibility study of a sensor-to-segment calibration method to enhance upper limb motion analysis using an IMU-based system for clinical and home environments
Source: PLoS One. 2025 Oct 24;20(10):e0334177. doi: 10.1371/journal.pone.0334177 (PMC12551884; doi:10.1371/journal.pone.0334177)
Supplement: S2 Table — (PDF) [file pone.0334177.s003.pdf]

**Table 1.** Detailed description of the tasks performed and the joint angles analysed

| Task | Description | Joint of interest |
|---|---|---|
| Draw | Grasp the pen<br>Draw following the path<br>Return the pen on the table | Shoulder<br>Elbow |
| Drink | Grasp the cup<br>Initial phase of the lifting<br>Lifting the cup to the mouth<br>Return the cup to the table | Elbow<br>Wrist |
| Move an object | Grasp the box<br>Move the box in the horizontal plane<br>Return the box on the table<br>Move the box to the shelf<br>Return the box on the table | Shoulder<br>Elbow |
| Unlock a locker | Grasp the key already inserted in the locker<br>Rotation of the key clockwise<br>Rotation of the key anticlockwise | Shoulder<br>Elbow |
