# Supplementary material for: Feasibility study of a sensor-to-segment calibration method to enhance upper limb motion analysis using an IMU-based system for clinical and home environments
Source: PLoS One. 2025 Oct 24;20(10):e0334177. doi: 10.1371/journal.pone.0334177 (PMC12551884; doi:10.1371/journal.pone.0334177)
Supplement: S1 File — Additionally, the rotation matrices used to define the angular position of the joint from the images were defined. (PDF) [file pone.0334177.s004.pdf]

Optoelectronic Motion Capture System

Table 1 describes the definition of the body segment analyzed for the left and right sides of the upper limb. Markers on the trunk body segment were placed on the seventh spinal process ($\mathbf{M}_{\text{C7}}$), deepest point of the incisura jugularis ($\mathbf{M}_{\text{IJ}}$) and the processus xiphoideus ($\mathbf{M}_{\text{PX}}$). Markers placed on the dorsal point on the acromioclavicular joint ($\mathbf{M}_{\text{ACR}}$) and on the most caudal point on the lateral and medial epicondyle ($\mathbf{M}_{\text{LEP}}$, $\mathbf{M}_{\text{MEP}}$) were used to define the upper arm segment, together with the middle point between $\mathbf{M}_{\text{LEP}}$ and $\mathbf{M}_{\text{MEP}}$, defined as $\mathbf{M}_{\text{M.Elb}}$. The position of the marker $\mathbf{M}_{\text{ACR\_R}}$ was adjusted according to [1]. The latter, together with the middle point ($\mathbf{M}_{\text{M.Wrs}}$) between the radial and ulnar styloid ($\mathbf{M}_{\text{RST}}$, $\mathbf{M}_{\text{UST}}$), and the radial styloid ($\mathbf{M}_{\text{RST}}$) were used to define the forearm segment. The hand segment was defined using $\mathbf{M}_{\text{M.Wrs}}$, $\mathbf{M}_{\text{RST}}$ and the 3rd metacarpal ($\mathbf{M}_{\text{MCP}}$). Seven IMUs were placed on the upper limb as follows: one sensor on the trunk, at the sternum level, one on the upper arm, centered on the lateral side of the proximal part, one on the forearm, at wrist level, and one on the dorsal surface of the hand.
See Fig.1 to know the placement of the markers.

Fig 1. The retroflective markers and inertial measurements units' placement site. Red circles represent the markers and the orange squares represent the sensors.

The rotations are described using Euler angles: the Y-X'-Y" for the shoulder, the Z-X'-Y" for the elbow and the Z-X'-Y" for the wrist, in accordance to the standards [2].

Segment	Origin	u	v	w
trs	$\mathbf{M}_{\text{JUG}}$	$\mathbf{v}_{\text{trs}}$ x $\mathbf{w}_{\text{trs}}$	$\frac{(\mathbf{M}_{\text{JUG}} - M_{\text{XP}})}{(\lVert \mathbf{M}_{\text{JUG}} - M_{\text{XP}} \rVert)}$	$\frac{(\mathbf{M}_{\text{JUG}} - M_{\text{C7}})}{(\lVert \mathbf{M}_{\text{JUG}} - M_{\text{C7}} \rVert)}$ x $\mathbf{v}_{\text{trs}}$
rua	$\mathbf{M}_{\text{M.Elb\_R}}$	$\mathbf{v}_{\text{rua}}$ x $\left(\frac{M_{\text{LEP\_R}} - \mathbf{M}_{\text{M.Elb\_R}}}{\lVert M_{\text{LEP\_R}} - \mathbf{M}_{\text{M.Elb\_R}} \rVert}\right)$	$\left(\frac{M_{\text{ACR\_R}} - \mathbf{M}_{\text{M.Elb\_R}}}{\lVert M_{\text{ACR\_R}} - \mathbf{M}_{\text{M.Elb\_R}} \rVert}\right)$	$\mathbf{u}_{\text{rua}}$ x $\mathbf{v}_{\text{rua}}$
rfa	$\mathbf{M}_{\text{M.Wrs\_R}}$	$\mathbf{v}_{\text{rfa}}$ x $\mathbf{w}_{\text{rfa}}$	$\left(\frac{\mathbf{M}_{\text{M.Elb\_R}} - \mathbf{M}_{\text{M.Wrs\_R}}}{\lVert \mathbf{M}_{\text{M.Elb\_R}} - \mathbf{M}_{\text{M.Wrs\_R}} \rVert}\right)$	$\left(\frac{M_{\text{RST\_R}} - \mathbf{M}_{\text{M.Wrs\_R}}}{\lVert M_{\text{RST\_R}} - \mathbf{M}_{\text{M.Wrs\_R}} \rVert}\right)$ x $\mathbf{v}_{\text{rfa}}$
rh	$\mathbf{M}_{\text{MCP\_R}}$	$\mathbf{v}_{\text{rh}}$ x $\mathbf{w}_{\text{rh}}$	$\left(\frac{M_{\text{M.Wrs\_R}} - \mathbf{M}_{\text{MCP\_R}}}{\lVert M_{\text{M.Wrs\_R}} - \mathbf{M}_{\text{MCP\_R}} \rVert}\right)$	$\left(\frac{M_{\text{RST\_R}} - \mathbf{M}_{\text{MCP\_R}}}{\lVert M_{\text{RST\_R}} - \mathbf{M}_{\text{MCP\_R}} \rVert}\right)$ x $\mathbf{v}_{\text{rh}}$

Table 1. Definition of the body segment coordinate systems for the right side

Proposed Picture-based calibration method

For the shoulder joint, the Y-X'-Y" order of rotation was chosen and the angles obtained are respectively α,β,γ as follow:

$$\alpha = \text{atan2d}\left(\frac{\mathbf{R}^{\text{tor}}_{\text{rua}}(1,2)}{\mathbf{R}^{\text{tor}}_{\text{rua}}(3,2)}\right) \beta = \text{atan2d}\left(\frac{(\mathbf{R}^{\text{tor}}_{\text{rua}}(3,2))^2 + (\mathbf{R}^{\text{tor}}_{\text{rua}}(1,2))^2}{(\mathbf{R}^{\text{tor}}_{\text{rua}}(2,2))^2}\right) \gamma = \text{atan2d}\left(\frac{\mathbf{R}^{\text{tor}}_{\text{rua}}(2,1)}{-\mathbf{R}^{\text{tor}}_{\text{rua}}(2,3)}\right) \quad (1)$$

For the elbow joint, the Z-X'-Y" order of rotation was chosen and the angles of the rfa respect to rua are respectively α,β,γ as follow:

$$\alpha = \text{atan2d}\left(\frac{-\mathbf{R}^{\text{rua}}_{\text{rfa}}(1,2)}{\mathbf{R}^{\text{rua}}_{\text{rfa}}(2,2)}\right) \beta = \text{atan2d}\left(\frac{(\mathbf{R}^{\text{rua}}_{\text{rfa}}(3,2))^2 + (\mathbf{R}^{\text{rua}}_{\text{rfa}}(3,1))^2}{(\mathbf{R}^{\text{rua}}_{\text{rfa}}(3,3))^2}\right) \gamma = \text{atan2d}\left(\frac{-\mathbf{R}^{\text{rua}}_{\text{rfa}}(3,1)}{\mathbf{R}^{\text{rua}}_{\text{rfa}}(3,3)}\right) \quad (2)$$

The same can be said for the angles of the rhand respect to the rfa, using the rotation matrix $\mathbf{R}^{\text{rfa}}_{\text{rhand}}$.

References

1. Rab G, Petuskey K, Bagley A. A method for determination of upper extremity kinematics. Gait and Posture. 2002;15(2):113–119. doi:10.1016/S0966-6362(01)00155-2.

2. Wu G, Helm FCTVD, Veeger HEJD, Makhsous M, Roy PV, Anglin C, et al. ISB recommendation on definitions of joint coordinate systems of various joints for the reporting of human joint motion — Part II : shoulder , elbow , wrist and hand. 2005;38:981–992. doi:10.1016/j.jbiomech.2004.05.042.
