# Supplementary material for: Feasibility study of a sensor-to-segment calibration method to enhance upper limb motion analysis using an IMU-based system for clinical and home environments
Source: PLoS One. 2025 Oct 24;20(10):e0334177. doi: 10.1371/journal.pone.0334177 (PMC12551884; doi:10.1371/journal.pone.0334177)
Supplement: S2 File — (PDF) [file pone.0334177.s005.pdf]

Upper limb joint kinematics

Table 1 reports the median(IQR) of both the offset, between the gold standard and the IMU data, and the RMSD obtained after removing the offset itself. Compared to the results presented in Table 2 of the Result section, this analysis shows a reduced discrepancy with respect to the OMC data, highlighting the contribution of systematic offset to overall error. These findings further support the importance of precise calibration procedures in enhancing the accuracy of kinematic data collected with an IMU-based system.

Table 1. Offset at the beginning of the recording and measures of disagreement (RMSD) between the OMC and each calibration method (IMU-AA, IMU-FA, IMU-AD, and IMU-PIC), reported as median (interquartile range) for the dominant hand. RMSD values were computed after removing the initial offset to isolate the effect of calibration on waveform similarity

		Shoulder			Elbow			Wrist		
Draw	Offset (deg)	Y-axis	X'-axis	Y”-axis	Z-axis	X'-axis	Y”-axis	Z-axis	X'-axis	Y”-axis
	IMU-AA	16 (87.59)	-2.22 (15.98)	-46.34 (71.89)	4.02 (17.78)	19.31 (20.64)	6.4 (17.42)	0.23 (14.1)	7.82 (20.65)	4.33 (8.87)
	IMU-FA	-9.47 (87.14)	20.93 (15.8)	19.67 (94.98)	7.67 (22.11)	4.54 (9.9)	7.74 (15.88)	1.82 (8.5)	-9 (35.27)	0.56 (4.89)
	IMU-AD	0.0 (0.0)	0.0 (0.0)	0.0 (0.0)	0.0 (0.0)	0.0 (0.0)	0.0 (0.0)	0.0 (0.0)	0.0 (0.0)	0.0 (0.0)
	IMU-PIC	-3.07 (14.47)	6.64 (8.1)	-3.42 (10.53)	-3.6 (8.24)	4.54 (9.9)	7.16 (6.59)	5.31 (6.69)	-1.47 (10.6)	0.56 (4.89)
	RMSD (deg)	Y-axis	X'-axis	Y”-axis	Z-axis	X'-axis	Y”-axis	Z-axis	X'-axis	Y”-axis
	IMU-AA	12.28 (19.91)	8.13 (6.25)	18.15 (19.69)	3.69 (6.29)	6.36 (6.55)	15.03 (8.91)	6.33 (5.4)	3.6 (2.88)	5.43 (4.19)
	IMU-FA	60.69 (99.48)	9.89 (17.46)	66.45 (102.99)	6.33 (9.62)	9.03 (10.07)	14.36 (10.8)	7.66 (12.81)	4.93 (4.06)	6.71 (7.29)
	IMU-AD	12.23 (7.79)	7.13 (6.22)	16.35 (7.91)	6.19 (8.8)	8.43 (4.27)	14.18 (12.28)	4.24 (11.2)	4.5 (5.28)	4.37 (3.76)
	IMU-PIC	10.63 (18.77)	4.63 (3.99)	15.12 (14.75)	8.92 (9.15)	7.7 (6.86)	14.41 (9.11)	7.1 (12.59)	4.97 (1.79)	6.08 (7.2)
Drink	Offset (deg)	Y-axis	X'-axis	Y”-axis	Z-axis	X'-axis	Y”-axis	Z-axis	X'-axis	Y”-axis
	IMU-AA	33.36 (63.01)	4.25 (14.89)	-36.42 (14.89)	1.54 (17.51)	12.82 (48.3)	10.98 (34.87)	-0.94 (12.46)	3.32 (22.2)	6.86 (36.56)
	IMU-FA	-3.51 (88.89)	21.52 (10.24)	7.41 (97.22)	4.19 (16.35)	2.72 (8.07)	9.45 (26.89)	-1.6 (14.01)	-14.87 (29.66)	-0.01 (3.6)
	IMU-AD	0.0 (0.0)	0.0 (0.0)	0.0 (0.0)	0.0 (0.0)	0.0 (0.0)	0.0 (0.0)	0.0 (0.0)	0.0 (0.0)	0.0 (0.0)
	IMU-PIC	7.07 (17.38)	8.45 (6.37)	-2.87 (24.84)	-6.3 (10.29)	2.72 (8.07)	4.24 (14.67)	-0.29 (5.71)	-1.52 (8.77)	-0.01 (3.6)
	RMSD (deg)	Y-axis	X'-axis	Y”-axis	Z-axis	X'-axis	Y”-axis	Z-axis	X'-axis	Y”-axis
	IMU-AA	27.42 (32.1)	6.93 (6.14)	28.05 (28.22)	6.77 (8.41)	10.02 (14.65)	17.68 (7.85)	7.35 (7.36)	7.62 (8.86)	10.48 (10.63)
	IMU-FA	72.36 (90.34)	8.22 (15.95)	69 (94.43)	10.88 (8.45)	10.74 (12)	16.93 (10.73)	9.05 (6.71)	8.53 (3.55)	11.23 (7.02)
	IMU-AD	14.28 (3.56)	6.32 (5.3)	12.2 (18.46)	12.3 (10.09)	8.93 (9.05)	17.36 (11.36)	12.68 (9.65)	7.01 (4.32)	11.73 (5.84)
	IMU-PIC	18.62 (22.11)	6.53 (7.48)	22.27 (29.97)	10.82 (8.83)	2.72 (12.38)	4.24 (12.49)	10.25 (8.2)	8.98 (3.44)	10.36 (7.27)
Move an object	Offset (deg)	Y-axis	X'-axis	Y”-axis	Z-axis	X'-axis	Y”-axis	Z-axis	X'-axis	Y”-axis
	IMU-AA	34.4 (51.43)	3.35 (9.53)	-20.36 (67.67)	7.73 (18.46)	5.26 (35.34)	2.05 (10.38)	1.34 (16.06)	2.78 (18.81)	7.99 (21.06)
	IMU-FA	-3.64 (81.56)	22.05 (13.3)	5.35 (111.03)	10.02 (17.91)	0.54 (8.39)	8.42 (23.64)	-0.99 (11.88)	-10.56 (43.72)	0.01 (4.96)
	IMU-AD	0.0 (0.0)	0.0 (0.0)	0.0 (0.0)	0.0 (0.0)	0.0 (0.0)	0.0 (0.0)	0.0 (0.0)	0.0 (0.0)	0.0 (0.0)
	IMU-PIC	2.28 (6.8)	9.02 (6.24)	-5.02 (15.67)	-4.88 (6.6)	0.54 (8.39)	-0.16 (7.58)	5.08 (11.21)	3.21 (14.09)	0.01 (4.96)
	RMSD (deg)	Y-axis	X'-axis	Y”-axis	Z-axis	X'-axis	Y”-axis	Z-axis	X'-axis	Y”-axis
	IMU-AA	34.76 (55.6)	8.47 (12.06)	32.49 (10.52)	12.99 (15.81)	13.62 (7.14)	25.47 (13.95)	13.35 (8.21)	10.09 (5.27)	21.21 (16.3)
	IMU-FA	89 (122.25)	12.36 (6.68)	89.96 (124.1)	18.04 (13.69)	13.66 (4.77)	22.12 (17.27)	17.64 (12.42)	10.05 (2.71)	19.8 (16.67)
	IMU-AD	12.2 (8.19)	10.92 (6.14)	20.25 (7.19)	20.5 (18.41)	13.39 (4.27)	22.42 (20.91)	10.39 (15.52)	10.71 (4.4)	22.13 (18.36)
	IMU-PIC	9.52 (11.39)	7.43 (7.96)	21.25 (18.9)	14.96 (18.37)	12.77 (5.32)	23.55 (16.48)	10.8 (20.53)	9.57 (5.79)	25.67 (19.79)
Unlock a locker	Offset (deg)	Y-axis	X'-axis	Y”-axis	Z-axis	X'-axis	Y”-axis	Z-axis	X'-axis	Y”-axis
	IMU-AA	7.44 (100.27)	2.44 (17.43)	-14.77 (98.44)	12.22 (17.47)	-2.89 (56.25)	-2.71 (23.55)	-9.79 (15.75)	3.88 (14.28)	-1.95 (12.98)
	IMU-FA	18.25 (90.58)	20.96 (8.44)	-17.71 (88.03)	3.97 (23.66)	0.47 (11.14)	10.12 (21.08)	0.35 (15.45)	-9.72 (33.66)	-0.13 (3.03)
	IMU-AD	0.0 (0.0)	0.0 (0.0)	0.0 (0.0)	0.0 (0.0)	0.0 (0.0)	0.0 (0.0)	0.0 (0.0)	0.0 (0.0)	0.0 (0.0)
	IMU-PIC	0.17 (16.18)	8.63 (4.34)	4.38 (23.65)	-3.99 (6.15)	0.47 (11.14)	6.33 (6.12)	1.08 (17.09)	2.59 (12.57)	-0.13 (3.03)
	RMSD (deg)	Y-axis	X'-axis	Y”-axis	Z-axis	X'-axis	Y”-axis	Z-axis	X'-axis	Y”-axis
	IMU-AA	28.62 (46.63)	7.56 (4.85)	26.43 (23.06)	10.55 (14.63)	9.63 (11.54)	24.22 (7.71)	5.55 (4.74)	5.6 (3.92)	15.03 (5.07)
	IMU-FA	57.74 (82.64)	9.68 (6.54)	65.77 (73.93)	10.13 (4.97)	11.97 (5.24)	21.99 (12.6)	8.63 (5.88)	6.35 (4.23)	14.29 (5.48)
	IMU-AD	13.78 (10.39)	8.34 (4.57)	18.68 (9.11)	9.37 (8.89)	11.91 (11.47)	23.04 (15.72)	9.19 (9.26)	5.54 (6.25)	15.11 (5.57)
	IMU-PIC	12.3 (12.1)	5.92 (3.02)	17.03 (22.33)	10.33 (5.25)	10.08 (7.71)	23.69 (12.9)	7.9 (6.77)	5.31 (4.19)	15.91 (5.78)
