# Supplementary material for: Feasibility study of a sensor-to-segment calibration method to enhance upper limb motion analysis using an IMU-based system for clinical and home environments
Source: PLoS One. 2025 Oct 24;20(10):e0334177. doi: 10.1371/journal.pone.0334177 (PMC12551884; doi:10.1371/journal.pone.0334177)
Supplement: S3 Table — (PDF) [file pone.0334177.s006.pdf]

Table 1. Range of motion recorded with the OMC system for the 10 subjects analyzed for the draw task.

ROM/Subj	Int/Ext Rot.	Elevation	Axial Rot	Flexion	CA	Pron./Sup	Flexion	Abduction	Pron./Sup
S02	135.27	20.18	112.09	92.5	17.76	32.53	21.55	28.7	8.68
S03	120.29	15.26	89.87	83.08	18.34	46.62	52.77	44.28	37.52
S04	92.83	47.4	67.96	44.8	17.96	29.96	18.01	29.47	4.57
S05	79.21	54.83	76.1	82.96	27.33	57.29	31.65	28.08	19.9
S07	156.21	40.74	130.93	75.16	9.39	28.8	29.28	25.51	5.69
S08	126.01	31.53	145.41	37.5	23.67	41.87	20.06	24	4.98
S12	29.16	17.01	21.64	25.02	17.75	23.79	11.31	21.25	4.52
S13	107.73	29.91	128.74	31.81	5.28	17.32	23.75	24.87	8.54
S14	114	18.54	93.69	53.38	49.8	43.94	15.5	30.98	10.42
S18	75.79	23.02	61.77	30.3	11.11	28.8	17.52	25.34	3.21
median	110.87	26.47	91.78	49.09	17.86	31.25	20.81	26.8	7.12
IQR	46.8	22.2	60.78	51.15	12.56	15.14	11.76	4.6	5.85

Table 2. Range of motion recorded with the OMC system for the 10 subjects analysed for the drink task.

	Int/Ext Rot.	Elevation	Axial Rot	Flexion	CA	Pron./Sup	Flexion	Abduction	Pron./Sup
S02	138.53	56.02	125.95	111.98	7.51	52.02	31.63	34.11	16.66
S03	134.38	17.19	116.36	80.34	16.32	33.66	32.48	40.24	8.74
S04	70.69	91.34	71.97	109.35	16.6	39.25	17.42	34.5	7.08
S05	112.72	70.55	103.56	131.12	28.37	59.97	31.96	32	13.91
S07	115.08	44.08	108.99	123.39	10.61	56.21	25.65	35.53	13.78
S08	112.24	37.7	101.04	103.31	22.1	54.2	38.11	31.87	10.72
S12	32.17	39.05	32.63	97.03	24.43	39.73	31.89	25.77	5.97
S13	131.73	63.58	130.54	75.04	9.98	43.07	16.17	28.31	4.84
S14	100.63	29.36	100.94	96.69	14.87	38.09	43.2	28.1	16.98
S18	85.17	40.71	92.25	111.86	15.07	76.3	36.78	60.69	16.99
median	112.48	42.4	102.3	106.33	15.7	47.55	31.93	33.06	12.25
IQR	46.56	25.88	24.11	15.29	11.49	16.96	11.13	7.22	9.58

Table 3. Range of motion recorded with the OMC system for the 10 subjects analysed for the move an object task.

	Int/Ext Rot.	Elevation	Axial Rot	Flexion	CA	Pron./Sup	Flexion	Abduction	Pron./Sup
S02	128.63	71.66	99.89	129.53	35.48	69.88	41.66	42.59	26.25
S03	111.16	58.01	114.92	128.6	16.49	73.81	33.7	45.6	26.88
S04	97.97	106.91	110.44	139.5	20.25	70.51	45.98	51.3	28.88
S05	107.06	117.24	103.42	133.07	19.09	67.89	48.25	78.56	29.46
S07	132.11	60.7	133.31	133.73	15.56	90.65	34.72	42.44	23.59
S08	118.18	63.43	210.9	75.98	21.89	61.24	30.57	84.28	24.12
S12	69.91	78.35	62.89	44.84	22.54	84.24	24.95	30.86	4.93
S13	118.46	83.67	114.55	120.66	14.41	48.48	32.66	59.24	13.78
S14	109	42.18	114.56	74.39	25.16	105.92	49.07	101.22	49.74
S18	114.17	85.25	110.69	64.7	14.86	77.29	28.21	39.04	16.75
median	112.67	75.01	112.62	124.63	19.67	72.16	34.21	48.45	25.19
IQR	11.4	24.55	11.5	58.68	6.98	16.35	15.41	36.12	12.13

Table 4. Range of motion recorded with the OMC system for the 10 subjects analysed for the unlock a locker task.

	Int/Ext Rot.	Elevation	Axial Rot	Flexion	CA	Pron./Sup	Flexion	Abduction	Pron./Sup
S02	137.98	65.55	126	129.47	14.13	140.44	51.58	39	26.74
S03	131.68	64.7	116.72	122.86	17.66	131.88	49.79	34.28	16.22
S04	110.05	93.99	97.49	128.27	14.96	132.91	61.1	30.76	16.11
S05	64.96	85.86	84.68	136.44	28.95	126.72	73.95	53.3	29.97
S07	151.36	71.17	150.63	114.84	16.55	120.4	31.52	26.86	8.61
S08	116.39	54.35	128.79	78.79	29.6	144.6	40.77	26.86	15.01
S12	21.32	62.59	90.6	48.36	26.47	135.85	22.2	36.05	2.95
S13	126.16	67.18	122.41	80.46	17.61	123.44	44.08	54.41	9.71
S14	114.11	38.58	104.57	79.82	14.91	132.47	38.69	20.96	12.97
S18	58.3	56.27	103.78	44.32	10.34	165.61	52.41	52.64	19.66
median	115.25	65.13	110.65	97.65	17.08	132.69	46.94	35.17	15.56
IQR	66.72	14.9	28.51	49.48	11.56	13.72	13.72	25.78	9.95
